# WISB: Warwick Integrative Synthetic Biology Centre

**DOI:** 10.1042/BST20160011

**Published:** 2016-06-09

**Authors:** John McCarthy

**Affiliations:** * School of Life Sciences, University of Warwick, Coventry CV4 7AL, U.K.

**Keywords:** engineering biosynthetic pathways, engineering microbial communities, engineering microbial effector systems in plants, Ethical, Legal and Societal Aspects (ELSA), RRI and outreach, postgraduate training, predictive biosystems engineering

## Abstract

Synthetic biology promises to create high-impact solutions to challenges in the areas of biotechnology, human/animal health, the environment, energy, materials and food security. Equally, synthetic biologists create tools and strategies that have the potential to help us answer important fundamental questions in biology. Warwick Integrative Synthetic Biology (WISB) pursues both of these mutually complementary ‘build to apply’ and ‘build to understand’ approaches. This is reflected in our research structure, in which a core theme on predictive biosystems engineering develops underpinning understanding as well as next-generation experimental/theoretical tools, and these are then incorporated into three applied themes in which we engineer biosynthetic pathways, microbial communities and microbial effector systems in plants. WISB takes a comprehensive approach to training, education and outreach. For example, WISB is a partner in the EPSRC/BBSRC-funded U.K. Doctoral Training Centre in synthetic biology, we have developed a new undergraduate module in the subject, and we have established five WISB Research Career Development Fellowships to support young group leaders. Research in Ethical, Legal and Societal Aspects (ELSA) of synthetic biology is embedded in our centre activities. WISB has been highly proactive in building an international research and training network that includes partners in Barcelona, Boston, Copenhagen, Madrid, Marburg, São Paulo, Tartu and Valencia.

Warwick Integrative Synthetic Biology (WISB) Centre seeks to deliver an integrated, internationally leading programme of research, innovation and training for synthetic biology. We are a multidisciplinary community comprising researchers with backgrounds in biology, chemistry, computer science, engineering, mathematics, physics and social science (Figure 1). WISB research and development explore multiple levels of biological organization, including molecules, circuits and pathways, cells, as well as multicellular and multi-species systems. The broad, but highly integrated, spectrum of WISB activities is outlined briefly in this short article. More information about the people and projects in WISB can be found on our website: www.wisb-uow.co.uk.

**Figure 1:**
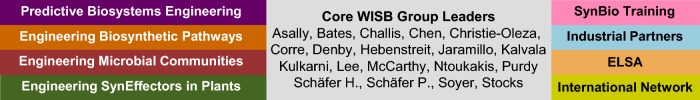
WISB: personnel and activities The core academic community comprises academics from the Departments of Life Sciences, Chemistry, Computer Science, Engineering and Education Studies. In addition, WISB comprises Associate Members from the University of Warwick, Honorary Members from other institutions, RCDFs, postdoctoral associates and PhD students and also trains and supports an iGEM team each year. More information can be found on our website: http://www.wisb-uow.co.uk.

## Predictive biosystems engineering

One of the most important goals for synthetic biology is to make system design having predictable consequences in terms of biological function. It is currently the case that assembling multiple (genetic) components together frequently generates unpredicted or suboptimal behaviour because we know too little about inter-component interactions or about circuitry–host interactions. Since this is not a satisfactory platform on which to base the future development of the field, we are using experimental and computational methods to develop new and improved types of biomolecular circuitry for both prokaryotic and eukaryotic hosts, and to develop advanced CAD tools to make this task faster, cheaper and easier. Rather than relying on the standard types of transcriptional part, we are constructing new types of post-transcriptional component and circuitry that rely, for example on control at the levels of mRNA translation and protein modification or that facilitate computation directly using DNA strand displacement. We are also seeking ways to improve the robustness and scalability of synthetic circuitry by optimizing system stochasticity and minimizing the negative effects of host–circuit interactions.

## Engineering biosynthetic pathways

Mankind has exploited microorganisms as sources of valuable metabolic products for millennia, but attempts to engineer biosynthetic pathways to create novel metabolic diversity are often hampered by insufficient understanding of underlying molecular mechanistic principles and can produce unpredictable outcomes. We aim to apply synthetic biology principles to develop a better understanding of biosynthetic pathways, which will enable more predictable pathway engineering. Computational design tools and yeast-mediated recombination are being applied to build refactored biosynthetic gene clusters for structurally complex metabolites with applications in medicine (e.g. antibiotics), agriculture (e.g. herbicides) and bioenergy. In parallel, orthogonal transcriptional and translational regulatory elements are being engineered with a view to fine-tuning expression from (refactored) biosynthetic gene clusters.

## Engineering microbial communities

Microbial species have generally evolved in the context of communities. There are many ways in which naturally evolved or engineered, microbial communities can benefit mankind, but this has been a relatively poorly explored aspect of synthetic biology. At WISB, we are pursuing a number of complementary approaches to advancing the understanding and benefits of engineered microbial communities. We are engineering synthetic microbial communities from the bottom up using (multiple) defined species that are functionally interlinked using metabolic, genetic and physical interactions, and reengineering existing communities using rational manipulations at the species and community level. Examples of the former category are projects on (i) three-species systems involving a phototroph that convert light into high-value chemicals in a closed ecosystem and (ii) a spatially organized community designed to perform environmental bioremediation. An example of the latter category is work on reengineering gut communities towards reducing production of TMAO, a by-product of a high-meat diet that has been shown to increase the risk of atherosclerosis.

## Engineering microbial effector systems in plants

Ensuring food security for the world's population is one of the greatest challenges faced by mankind. WISB researchers are developing new types of synthetic control system that use synthetic effectors (SynEffectors), derived from natural effectors of plant pathogens and mutualists. Natural microbial effectors are targeted to bespoke pathways within plants in order to engineer (orthogonal) temporal and spatial control of plant responses, thus paving the way for the development of plants with enhanced resistance to stress and microbial attack. A key part of this work is the identification of effectors in pathogenic and mutualistic microbes that control developmental (senescence), immunity and abiotic stress (drought, high-light) pathways. Particular emphasis is given to mechanisms that can uncouple growth and immunity–pathways that typically act antagonistically. Synthetic versions of these effectors (SynEffectors) will be engineered into new plant regulatory systems.

## Training and education in synthetic biology

WISB is committed to delivering a comprehensive and coherent programme of excellent education and training in synthetic biology in collaboration with academic and industrial partners. This starts with dedicated teaching elements (including a third-year module) at the undergraduate level that provides the platform for students to pursue the Master's degree (MBio) in synthetic biology in their fourth year of study. Each year, WISB academics train and support a team that competes in the international Genetically Engineered Machine (iGEM) competition. WISB is a partner in the U.K.'s only Centre of Doctoral Training in Synthetic Biology. We also provide extensive skills training for postdoctoral researchers working in synthetic biology and support a cohort of WISB Research Career Development Fellows (RCDFs). All WISB members benefit from bespoke training courses on transferable skills and on enterprise and impact. Moreover, the WISB community pursues an extensive and diverse programme of outreach and public engagement.

## Ethical, Legal and Societal Aspects of synthetic biology

We believe that WISB can most effectively explore, and where appropriate implement principles related to Ethical, Legal and Societal Aspects (ELSA) of synthetic biology in the context of collaboration and partnership with other centres, both national and international. WISB is accordingly a member of the U.K. National Synthetic Biology network. We are also working together with the Department of Media, Cognition and Communication at the University of Copenhagen to develop new research and to deliver training courses, on science and society.

## National and international partnerships

WISB is building strong partnerships in research and training with academic institutions across the globe. We collaborate in multiple areas with colleagues in the other U.K. Synthetic Biology Research Centres (SBRCs). In addition, our international partners already include the Biomass Systems and Synthetic Biology Centre (BSSB), São Paulo, Brazil; Boston University BioDesign Center; Experimental and Health Sciences, Universitat Pompeu Fabra, Barcelona, Spain; Löwe Centre for Synthetic Microbiology (SYNMIKRO), Philipps-Universität Marburg, Germany; SynBio Centre, Tartu University, Estonia; the Synthetic Biology Centre at the University of Copenhagen. This network is expected to be mutually beneficial for all partners, allowing us all to enhance the breadth and scale of our research and training activities. WISB is also collaborating with a range of companies, currently including Abolis, Algenuity, BASF, Biopolis, Evolva, Green Biologics, Ingenza, Isomerase, Microsoft Research, Syngenta and Tata Steel.

## WISB facilities

With funding from the BBSRC and EPSRC, WISB has established a state-of-the-art Research Technology Facility designed to support a diversity of synthetic biology projects, core staff to help manage its activities and a large cohort of postdoctoral researchers and PhD students. The instrumentation/technologies in the RTF include wide-field and spinning disk confocal microscopy, flow cytometry, cell sorting, microfluidics, surface plasmon resonance, LC–MS, ion chromatography, HPLC, 3D printers, micro- and macro-scale bioreactors, chemostats, gas analyser and robotics.

## Relevant websites

1. WISB (http://www.wisb-uow.co.uk)
2. Synthetic Biology CDT (www.warwick.ac.uk/synbiocdt)

## Abbreviations

BBSRC: Biotechnology and Biological Sciences Research Council
CAD: computer-aided design
ELSA: Ethical, Legal and Societal Aspects
EPSRC: Engineering and Physical Sciences Research Council
iGEM: international Genetically Engineered Machine
RCDF: Research Career Development Fellow
RRI: responsible research and innovation
RTF: Research Technology Facility
TMAO: trimethylamine N-oxide
WISB: Warwick Integrative Synthetic Biology
